# Characterization and anti-uterine tumor effect of extract from *Prunella vulgaris* L.

**DOI:** 10.1186/s12906-020-02986-5

**Published:** 2020-06-18

**Authors:** Yan Lin, Chao Yang, Jie Tang, Chun Li, Zhi-min Zhang, Bo-hou Xia, Ya-mei Li, Qing-zhi He, Li-mei Lin, Duan-fang Liao

**Affiliations:** 1 Key Laboratory for Quality Evaluation of Bulk Herbs of Hunan Province, College of Pharmacy, Hunan University of Chinese Medicine, No.300 Xueshi Road, Changsha, 410208 People’s Republic of China; 2 China Institute of Chinese Materia Medica, China Academy of Chinese Medical Sciences, Beijing, 100700 People’s Republic of China; 3 Hunan Province Cooperative Innovation Center for Molecular Target New Drug Study, School of Pharmacy and Life Science, University of South China, Hengyang, 421001 People’s Republic of China

**Keywords:** *Prunella vulgaris* L., Uterine Myoma, GC-MS, Apoptosis

## Abstract

**Background:**

The flowers and dried fruit spikes of *Prunella vulgaris* L. (*P. vulgaris* L.) have been widely used in traditional Chinese medicine and food. *P. vulgaris* L. is regarded as a good option for treating uterine myoma (UM). However, scientific evidence of anti-UM activity of the extract of *P. vulgaris* L. (PVE) is lacking. The present study aimed to characterize the chemical composition of PVE and evaluate the pharmacodynamics and mechanism of PVE against UM.

**Methods:**

The chemical composition of PVE was analyzed by GC-MS. MTT was used to screen and evaluate cell proliferation and toxicity. Double fluorescence flow cytometry method were used to determine the apoptosis and cell cycle progression of UM cells under PVE treatment. The anti-UM activity of PVE was investigated by using a specific-pathogen-free (SPF) rat model of UM. TUNEL staining was used to detect the apoptosis of UM cells. The concentrations of estrogen and progesterone in the serum of SPF rats were detected by ELISA. The expression levels of PCNA, estrogen receptor alpha, estrogen receptor beta, progesterone receptor, survivin, caspase-3, Bax and Bcl-2 in the uterus of SPF rats was detected by immunohistochemistry (IHC).

**Results:**

The extraction rate of PVE was 8.1%. The main components were squalene (28.3%), linoleic acid (9.96%), linolenic acid (9.95%), stearic acid (6.26%) and oleic acid (5.51%). In vitro, PVE had significant anti-human UM cell activity, exhibited no drug toxicity, promoted the apoptosis of human UM cells, and inhibited the transition of UM cells from the G0/G1 stage into the G2 stage, in which DNA replication occurs. In vivo, PVE had significant anti-UM activity. PVE decreased the concentrations of estrogen and progesterone and downregulated the expression levels of the estrogen and progesterone receptors through the estrogen signaling pathway. PVE also promoted the apoptosis of UM cells by downregulating the expression levels of the survivin and Bcl-2 proteins and upregulating the expression levels of caspase-3 and Bax through the mitochondria-mediated apoptotic pathway.

**Conclusion:**

PVE has marked anti-UM activity. PVE can be used as an ideal candidate drug to treat UM.

## Background

Uterine myoma (UM) is the most frequent benign tumor in women and is responsible for menorrhagia, other forms of abnormal uterine bleeding, iron deficiency anemia, and urinary incontinence [1]. The pathogenesis of UM may be related to sex hormones and their receptors, cell proliferation and apoptosis, abnormal signal transduction pathways, molecular genetics and other factors [2, 3]. The tumor growth rate of UM is associated with the estrogen signaling pathway [4]. Estrogen and progesterone can directly or indirectly affect the growth and development of UM [5]. Estrogen and progesterone can regulate the expression of the growth factors of UM [6, 7]. The inhibitory effects of estrogen and progesterone on UM tumor growth are mainly mediated by suppressing the expression levels of the receptors of these hormones [8]. The apoptosis of UM cells is associated with the mitochondria-mediated apoptotic pathway, which shifts the balance in the Bcl-2 family toward the proapoptotic members, such as Bax [9]. Bcl-2 protein located on mitochondrial membranes suppressed the release of apoptogenic proteins from mitochondria to the cytosol. Mitochondria-mediated apoptosis is also mediated by the activation of Bax, which results in an increase in caspase-3 [10]*.* The intrinsic mitochondria-mediated apoptotic pathway is also triggered by downregulating Bcl-2 protein expression, which activates caspase-3, the final executioner of apoptosis [11]. UM is commonly treated by corticosteroids, which are associated with a high recurrence rate and marked side effects, and by hormone therapy, which induces substantial pain [12]. Traditional Chinese Medicine has a definite advantage in curing UM [13].

The flowers and dried fruit spikes of *Prunella vulgaris* L. (*P. vulgaris* L.) have been widely used in traditional Chinese medicine and food. This plant has antitumor, anti-inflammatory, antiviral, antioxidant, and antibacterial functions [14]. Moreover, *P. vulgaris* L. is regarded as a good option for treating constipation, mammary gland hyperplasia, hysteromyoma, oophoritis cysts and breast cancer [15–17]. It is rich in essential oil, triterpenes and polysaccharide constituents. The essential oil of this plant has an inhibitory effect on the proliferation of cervical cancer and breast cancer cells [18–20]. Triterpenes have an inhibitory effect on the proliferation of breast cancer cells and can inhibit the estrogen receptor signaling pathway [21–23]. Polysaccharides have an inhibitory effect on the proliferation of breast cancer cells and can induce apoptosis [24].

In our previous research, we found that among the extract of *P. vulgaris* L. obtained by supercritical fluid (CO_2_) extraction and its triterpene and polysaccharide fractions, the extract was most effective for treating UM. The triterpenes were the most effective fraction for treating breast cancer, and the polysaccharides were the most effective fraction for the treatment of lipid disorders. Therefore, in the present study, the extract of *Prunella vulgaris* L. (PVE) was extracted by supercritical fluid (CO_2_) extraction and characterized by GC-MS, and its effects against UM and the underlying mechanisms were investigated. We investigated, for the first time, the effects of PVE on the growth and apoptosis of UM cells. The apoptotic cell death of UM induced by PVE was confirmed in vitro by the MTT method and double fluorescence flow cytometry method and in vivo by hematoxylin and eosin (HE) staining, TUNEL staining, ELISA, and immunohistochemistry (IHC).

## Methods

### Plant material

Flowers and dried fruit spikes of *P. vulgaris L.* were purchased in Anhui province and confirmed as *P. vulgaris L.* by Limin Gong, an Associate Professor in the Pharmacy Teaching Department at the Hunan University of Chinese Medicine.

### Cells

Human uterine smooth muscle cells (HUSMCs) and human uterine myoma cells (HUMCs) (SK-UT-1) were purchased from the Cell Bank of the Chinese Academy of Science (Shanghai, China).

### Antibodies and reagents

The proliferating cell nuclear antigen (PCNA) kit, TUNEL kit, caspase-3 antibody, Bax antibody, Bcl-2 antibody, survivin antibody, estrogen receptor alpha (ER-α) kit, estrogen receptor beta (ER-β) kit, progesterone receptor (PR) kit, estrogen kit, progesterone kit, and HE stain used in this study were purchased from Beijing Biosynthesis Biotechnology Co., Ltd. (Beijing, China). Progesterone, Gong Liu-qing capsule (GLC), estradiol benzoate, and mifepristone (MF) were purchased from the First Hospital of Hunan University of Chinese Medicine (Changsha, China). 3-(4,5-Dimethyl-2-thiazolyl)-2,5-diphenyl-tetra-zolium bromide (MTT) was purchased from Sigma Chemical Company (St. Louis, USA), and Dulbecco’s modified Eagle’s medium (DMEM) was purchased from HyClone (Logan, UT, USA). Fetal bovine serum (FBS) was purchased from Gibco Company (Grand Island, NY, USA). An apoptosis kit and cell cycle kit were purchased from Multi Sciences (Lianke) Biotech, Co., Ltd. (Hangzhou, China).

### Preparation and analysis of PVE

PVE was extracted for 120 min at 25 MPa and 30 °C by supercritical fluid (CO_2_) extraction. One milliliter of PVE was accurately measured and dissolved in ethyl acetate to a final volume of 10 ml in a volumetric flask. PVE components were determined by GC-MS. A DB-5MS quartz capillary column (30 m × 0.25 mm × 0.25 μm, Agilent J&W Scientific, Folsom, USA) was used. The injector temperature was set to 280 °C. A total of 1 μl of sample was injected, with a split ratio of 1:1. The flow rate of helium carrier gas (99.999%) was set to 1.0 ml/min. The temperature was first set at 100 °C, held for 2 min, gradually increased at a column temperature rate of 5 °C/min to 270 °C and held for 5 min. The temperate of the electron ionization source was 200 °C. The solvent delay duration was 6.5 min, and the MS scanning range was 35–550 m/z. The chromatograms were subjected to ion-pair extraction, peak alignment, peak matching, and peak amplitude correction using NISTOS and NISTOSs.

### Cell experiments

#### Toxicity evaluation of HUSMCs and HUMCs

HUSMCs and HUMCs in the logarithmic growth phase were separately plated in 96-well plates at a density of 1.0 × 10^4^ cells/well. The edge wells were filled with sterile PBS. The cells were cultured at 37 °C in a 5% CO_2_ atmosphere for 24 h. The culture medium in the wells was discarded, and the cells were treated with a PVE concentration of 1.0, 2.0, 3.0, 4.0, 5.0, 6.0, 7.0 or 8.0 mg/ml and a GLC concentration of 1.0, 2.0, 3.0, 4.0, 5.0, 6.0, 7.0, 8.0 or 9.0 mg/ml. A negative control group and a positive control group were included in parallel. Each concentration was represented in five parallel wells. After stimulating the cells with the appropriate drugs for 24 h, the culture medium was discarded. Next, 100 μl of 0.5 mg/ml MTT was added to each well, and the cells were incubated for 4 h. The culture medium was discarded, and 150 μl of DMSO was added to each well and incubated for 10 min on a horizontal shaker. When the violet crystals were completely dissolved, the absorbance at 490 nm (A_490_) was measured using a microplate reader (Thermo Electron Corporation, USA). The rates of cell growth inhibition and the IC50 were then calculated.

#### Detection of apoptosis and cell cycle in HUMCs

HUMCs in the logarithmic growth phase were seeded into 6 culture flasks at a density of 5.0 × 10^5^ cells/well. The cells were cultured at 37 °C in a 5% CO_2_ atmosphere for 24 h. The culture medium in the wells was then discarded. Subsequently, the cells were washed twice with PBS and treated with 5 ml PVE at a concentration of 0.65 (0.25 × IC50), 1.30 (0.50 × IC50), 1.95 (0.75 × IC50), or 2.60 mg/ml (1.0 × IC50) and GLC at a concentration of 4.65 mg/ml. After stimulating the cells with the drugs for 24 h, the cells were centrifuged at 1000 rpm for 3 min. The culture medium was then discarded. Next, the cells were washed twice with PBS, centrifuged, and collected. The cells were suspended in Annexin V buffer solution, and 5 μl of each of Annexin V-FITC and propidium iodide (PI) was added to each well. The cells were then incubated for 15 min in the dark. The cell cycle was measured by double fluorescence flow cytometry.

### Animal experiment

#### Animal

Specific-pathogen-free (SPF) female rats (200 ± 20 g) were purchased from Hunan SJA Laboratory Animal Co., Ltd. (Changsha, China). The animal permit number was SCXK (Xiang) 2016–0002. The temperature and humidity used for animal housing met the requirements for housing experimental animals. The rats were maintained under a 12 h dark-light cycle and were acclimated for 1 week prior to the experiment. Animal care and treatments were conducted according to guidelines and protocols approved by the Animal Care and Use Committee of the University of Hunan University of Chinese Medicine (Changsha, China). All efforts were made to minimize the number of animals and their suffering.

#### Model construction and pharmacological intervention

A total of 70 rats were randomly divided into the following 7 groups (*n* = 10 per group): control group (C); model group (M); GLC positive control group (GLC, an antiprogesterone drug, has been widely used to treat UM, 0.30 g/kg [25]); mifepristone positive control group (mifepristone has been widely used to treat UM, 5.4 mg/kg [26]); and low-, medium-, and high-dose PVE groups (LPVE (0.11 g/kg), MPVE (0.22 g/kg), and HPVE (0.44 g/kg), respectively). The control group was treated with normal saline by oral gavage, and the other groups were treated with estradiol benzoate injection (0.4 mg) and progesterone injection (0.2 mg) every other day for 60 days [27]. After the model establishment, the control and model groups were treated with normal saline by oral gavage, and the other groups were treated with the indicated medicine by oral gavage. Oral administration was carried out every morning for 30 days. At the end of the treatment, rats were injected with a high concentration of pentobarbital sodium (80 mg/kg) for anesthesia, blood was collected from the abdominal aorta, and cervical dislocation was performed. The uteri were collected and weighed. Serum samples were prepared by centrifuging the collected blood samples (at 2000 rpm for 10 min at 4 °C) and then stored at − 80 °C.

#### HE staining of UM

The uterine tissue samples were fixed in 10% formalin. Then, the paraffin-embedded tissue samples were sectioned into 5 μm-thick slices. The sections were stained with HE and examined under a light microscope (Model IX71, Olympus, Tokyo, Japan).

#### Detection of estrogen and progesterone

To detect the effect of PVE on estrogen content following estradiol benzoate and progesterone treatment, the concentrations of estrogen in the serum samples were detected by using the estrogen ELISA Kits (Beijing Biosynthesis Biotechnology Co., Beijing, China) according to the mamufacture’s instructions. The concentrations of estrogen were calculated from a standard curve and the results are expressd in pg/ml. To detect the effect of PVE on progesterone content, the serum concentrations of progesterone were detected by using the progesterone ELISA Kits (Beijing Biosynthesis Biotechnology Co., Beijing, China) according to the mamufacture’s instructions. The concentrations of progesterone were calculated from a standard curve and the results are expressd in ng/ml.

#### Detection of apoptosis in UM

Uterine tissue samples were collected, and the extent of cell apoptosis in the UM was measured using the TUNEL kit. The cell apoptosis assay stained in the green channel. DAPI was applied as a nuclear counter-stain in the blue channel. Images were taken with light microscopy. The TUNEL assay figures were analyzed by Image-Pro Plus (version 6.0) software (Media Cybernetics Inc., Maryland, USA). TUNEL-positive cells were considered to be undergoing apoptosis. The proportion of apoptotic uterine tissue was determined by dividing the number of TUNEL-positive nuclei by the total number of nuclei.

#### IHC of UM

Antibodies against PCNA, caspase-3, Bax, Bcl-2, survivin, ER-α, ER-β and PR were used to detect the expression levels of PCNA, caspase-3, Bax, Bcl-2, survivin, ER-α, ER-β and PR in uterine tissues through IHC. The proportion of positive expression in uterine tissue was determined by dividing the number of positive cells by the total number of cells.

### Statistical analysis

The data were represented as mean ± SD and analyzed by SPSS 13.0 software (SPSS Inc., Chicago, USA). Student’s *t*-test was used for comparisons between groups. One-way analysis of variance (ANOVA) was used for comparisons among groups. Nonparametric data were analyzed by Mann-Whitney U test. *p* < 0.05 was considered statistically significant.

## Results

### Chemical composition of PVE

The extraction rate of PVE achieved by the supercritical fluid (CO_2_) extraction kit was 8.1%. Sixteen components were identified in PVE by GC-MS. The major constituents were squalene (28.03%), linoleic acid (9.96%), α-linolenic acid (9.95%), octadecanoic acid (6.26%), and oleic acid (5.51%) (Table 1).

**Table 1:** The composition of the extract in *Prunella vulgaris* L.

No	t_R_ (min)	Compouds	Chemical formula	Relative molecular mass	Relative contents (%)	Similarity (%)
1	4.762	m-Dimethylbenzene	C_8_H_10_	106	0.14	98
2	9.613	D-Limonene	C_10_H_16_	136	0.13	96
3	26.268	2-Hydroxy-4-methoxy acetophenone	C_9_H_10_O_3_	166	1.07	98
4	28.802	Pentadecane	C_15_H_32_	212	0.19	94
5	32.537	Hexadecane	C_16_H_34_	226	0.19	96
6	36.103	Heptadecane	C_17_H_36_	240	0.53	97
7	42.759	n-Heneicosane	C_21_H_44_	296	0.48	96
8	45.670	Palmitic acid	C_16_H_32_O_2_	256	2.37	93
9	51.184	Linoleic acid	C_18_H_32_O_2_	280	9.96	89
10	51.450	α-Linolenic acid	C_18_H_30_O_2_	278	9.95	90
11	51.570	Oleic Acid	C_18_H_34_O_2_	282	5.51	93
12	51.837	Octadecanoic acid	C_18_H_36_O_2_	284	6.26	94
13	56.450	Arachidic acid	C_20_H_40_O_2_	312	2.71	91
14	60.310	1,2-Benzenedicarboxylic acid	C_24_H_38_O_4_	390	2.76	96
15	67.367	Squalene	C_30_H_50_	410	28.03	95
16	70.237	n-Tetratriacontane	C_34_H_70_	478	2.15	94

### Toxicity evaluation in HUSMCs and HUMCs

**Fig. 1:**
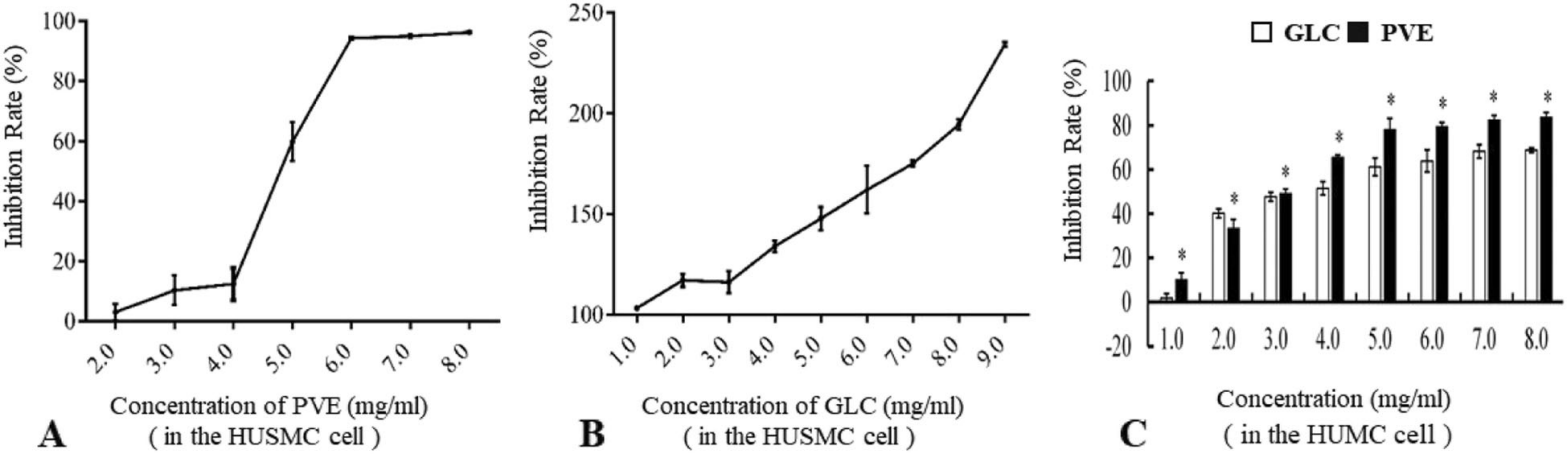
Inhibition rates of PVE and GLC by MTT assay. **a**: Inhibition rates of treatment with varying PVE concentrations of 2.0, 3.0, 4.0, 5.0, 6.0, 7.0 or 8.0 mg/ml at 24 h in the HUSMC cells; **b**: Inhibition rates of treatment with varying GLC concentrations of 1.0, 2.0, 3.0, 4.0, 5.0, 6.0, 7.0, 8.0 or 9.0 mg/ml at 24 h in the HUSMC cells; **c**: Inhibition rates of treatment with varying PVE or GLC concentrations of 1.0, 2.0, 3.0, 4.0, 5.0, 6.0, 7.0 or 8.0 mg/ml at 24 h in the HUMC cells, compared with the GLC group, *p < 0.05

The toxicities of PVE and GLC to HUSMCs were assessed by MTT staining. HUSMCs were treated with various concentrations of PVE and GLC. Toxicity curves were constructed with concentration on the X-axis and inhibition rate on the Y-axis and are shown in Fig. 1a-b. In HUSMCs, the IC50 of PVE was 4.58 mg/ml, whereas GLC did not show any toxicity. In HUMCs, the IC50 of PVE was 2.60 mg/ml, while the IC50 of GLC was 4.65 mg/ml. The inhibitory rate increased with PVE concentration. Moreover, the inhibition of proliferation induced by PVE was significantly increased compared with that induced by the positive control drug (GLC) (*p* < 0.05) (Fig. 1c).

### Detection of apoptosis and cell cycle in HUMCs

**Fig. 2:**
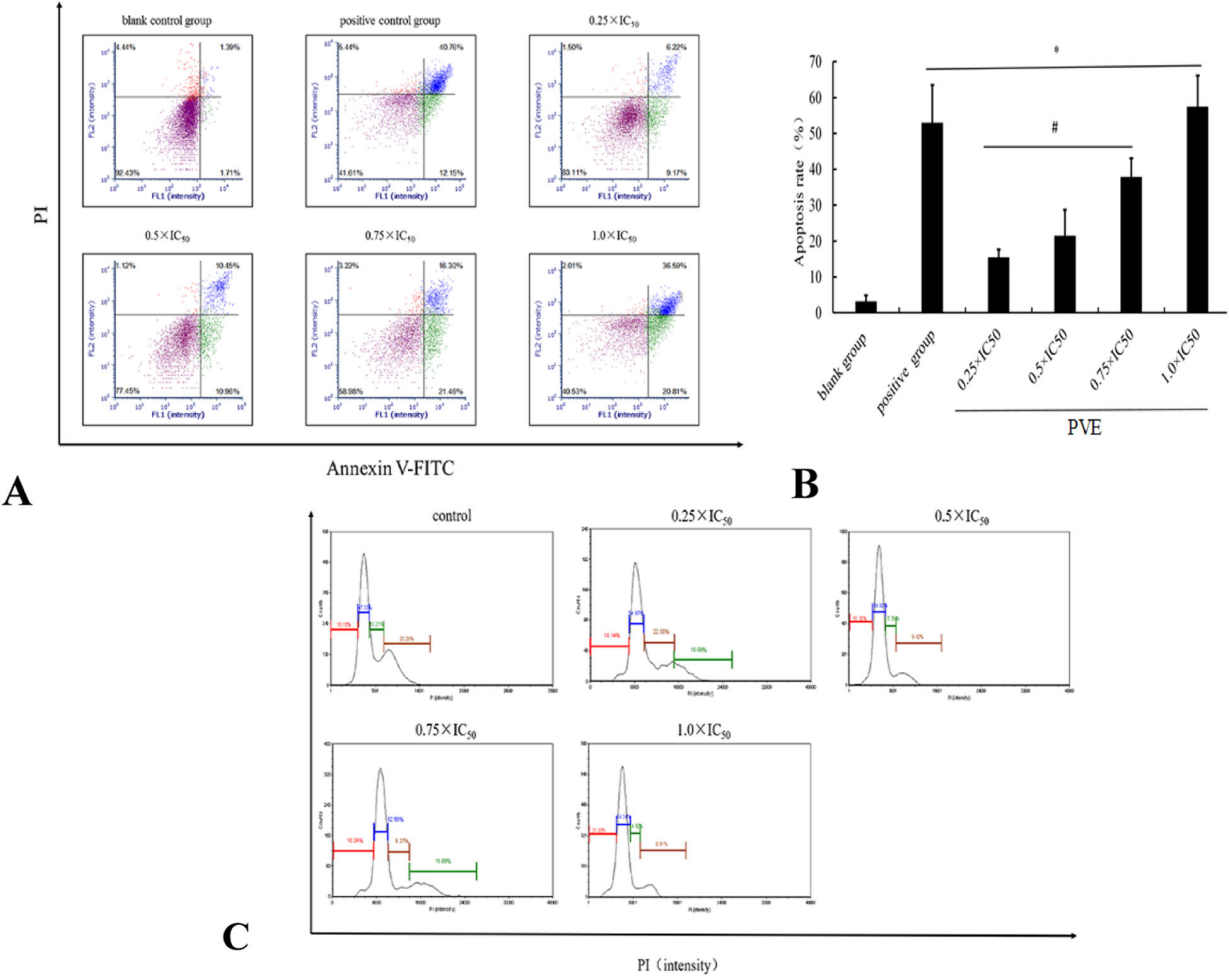
Apoptosis and cell cycle of PVE and GLC by cell flow cytometry analysis. The HUMC Cells were treated with varying PVE concentrations of 0, 0.25, 0.5, 0.75, and 1.0 × IC50 (2.6 mg/ml) or GLC concentrations of 4.65 mg/ml. After 24 h treatment, the cells were analyzed by Annexin V and PI staining. Percentages of cells in both early and late apoptosis were counted (**a**: apoptosis; **b**: apoptosis rate, compared with the blank group, ^#^*p* < 0.05, compared with the GLC group, *p < 0.05; **c**: cell cycle)

Because the IC50 of PVE in HUSMCs was 4.58 mg/ml and GLC did not show any toxicity, the apoptosis of HUMCs induced by PVE and GLC was assessed only by double fluorescence flow cytometry method. The percentage of apoptotic cells was 57.40% with PVE treatment and 52.91% with GLC treatment (Fig. 2a-b). These results indicated that cell apoptosis occurred. Compared with the model group, the PVE treatment groups and GLC treatment group exhibited significantly inhibited proliferation (*p* < 0.05), demonstrating that treatments with different concentrations of PVE and treatment with GLC had inhibitory effects. The GLC and PVE (1.0 × IC50) treatments had comparable effects. The cell cycle was detected in the different groups (Fig. 2c). The time in the G0/G1 phase increased with increasing PVE concentration. In addition, the cell cycle of HUMCs treated with PVE was arrested at the G0/G1 stage. Thus, the proliferation of HUMCs was inhibited at the G2 stage, precluding DNA synthesis (Table 2).

**Table 2:** Cell cycle distribution treated with PVE at different concentrations in the HUMC cell

Cell cycle	Concentration	Cell cycle distribution
G0-G1	0	47.33%
	0.25 × IC_50_	54.97%
	0.5 × IC_50_	62.90%
	0.75 × IC_50_	64.34%
	1.0 × IC_50_	69.00%
G2	0	20.28%
	0.25 × IC_50_	15.69%
	0.5 × IC_50_	10.69%
	0.75 × IC_50_	8.91%
	1.0 × IC_50_	9.42%

### Detection of UM weights

**Fig. 3:**
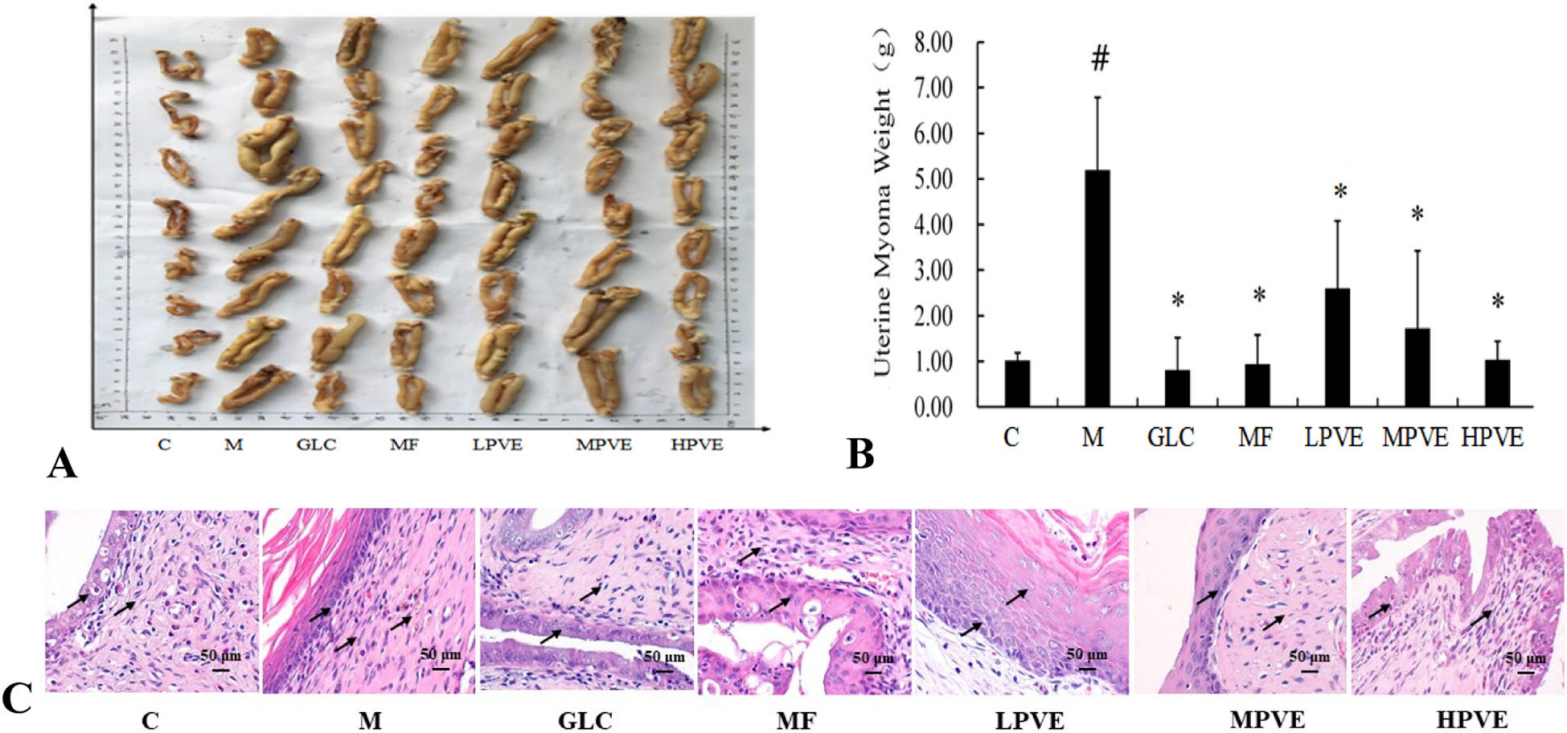
Effects of PVE on uterus volume, uterine myoma weights and uterus histology. The groups are shown as follows: control (C); model (M); GLC positive control group (GLC, 0.30 g/kg); mifepristone positive control group (MF, 5.4 mg/kg); low-dose PVE (LPVE, 0.11 g/kg); medium-dose PVE (MPVE, 0.22 g/kg); high-dose PVE (HPVE, 0.44 g/kg). Rats were treated with estradiol benzoate injection (0.4 mg) and progesterone injection (0.2 mg) every other day for 60 days for model construction. Model groups were treated with normal saline, and the other groups were treated with the indicated medicine by oral gavage for 30 days. At the end of the treatment, the uteri were collected and weighed. The uterine tissue samples were stained with HE. **a** uterus volume; **b** uterus myoma weight, compared with the control group, ^#^*p* < 0.05, compared with the model group, *p < 0.05; **c** uterus histological changes by HE staining (arrow), × 400 magnification, scale bar:50 μm

UM weight was significantly different between the model group and control group (*p* < 0.05). In addition, UM weight was significantly different between the model group and each of the groups treated with GLC; mifepristone; and high, medium, and low doses of PVE (*p* < 0.05), indicating that these drugs can inhibit the growth of UM (Table 3, Fig. 3a-b). The GLC-treated group, mifepristone-treated group, high-dose PVE-treated group, and the medium-dose PVE-treated group exhibited similar extents of inhibited UM growth.

**Table 3:** Tumor suppressor rate of the rats in the different groups

Group	Tumor suppressor rate(%)
Control	/
Model	/
GLC	84.32
Mifepristone	81.94
Low-dose PVE	49.80
Medium-dose PVE	66.72
High-dose PVE	80.04

### HE staining of UM

As shown in Fig. 3c, uterine swelling, uterine mass, hemorrhagic damage, and uterine retention were increased in the model group compared with the control group. Moreover, compared with the control group, the model group exhibited increased proliferation of endometrial epithelial cells, elongated and enlarged uterine smooth muscle, and increased eosinophils. These results showed that the UM model was successfully established. Compared with the model group, the groups treated with GLC, mifepristone, and a high dose of PVE showed improved histological appearance, as evidenced by decreasing uterine damage and reduced or absent edema. The uterus returned to the normal condition with PVE treatment. The results showed that PVE at a high dose can be used to successfully treat UM (Fig. 3c).

### Detection of estrogen and progesterone in serum

**Fig. 4:**
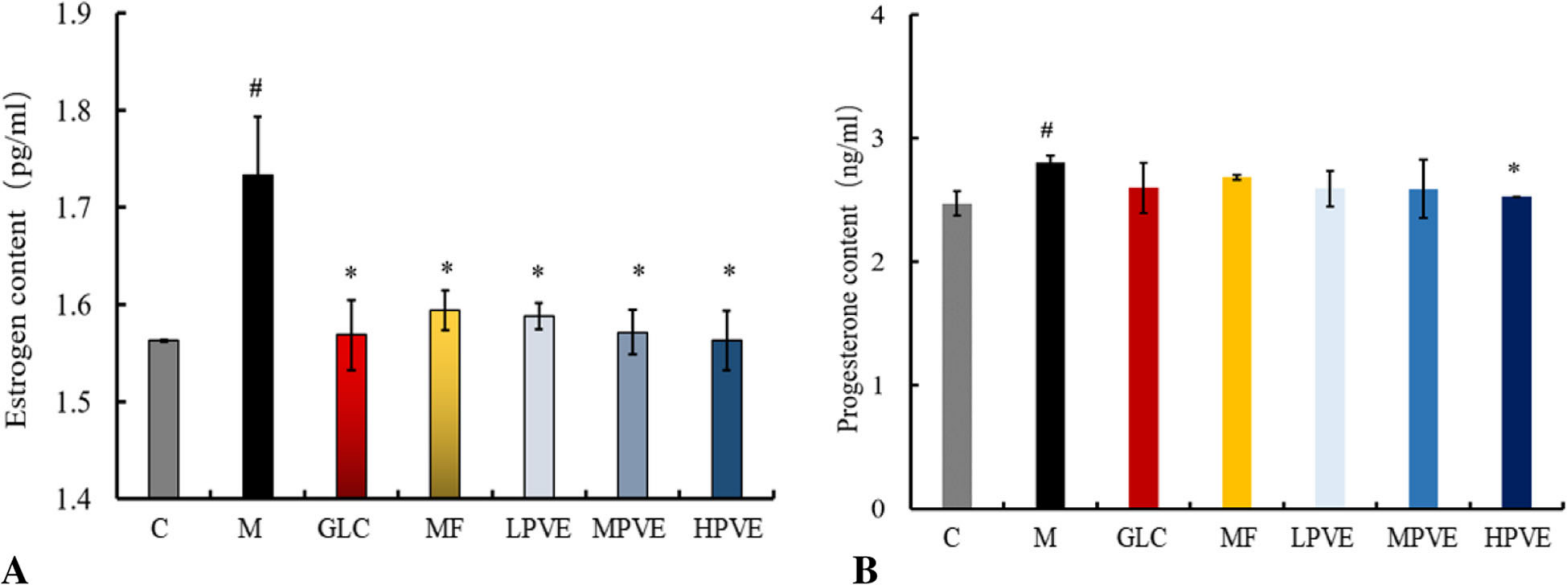
Effects of PVE on estrogen and progesterone content following estradiol benzoate and progesterone treatment. The groups are shown as follows: control (C); model (M); GLC positive control group (GLC, 0.30 g/kg); mifepristone positive control group (MF, 5.4 mg/kg); low-dose PVE (LPVE, 0.11 g/kg); medium-dose PVE (MPVE, 0.22 g/kg); high-dose PVE (HPVE, 0.44 g/kg). **a**: the content of estrogen detected in the serum by the estrogen ELISA Kits; **b**: the content of progesterone detected in the serum by the progesterone ELISA Kits; compared with the control group, ^#^*p* < 0.05; compared with the model group, *p < 0.05)

As shown in Fig. 4, the estrogen and progesterone concentrations were significantly increased in the model group, compared with the control group. These results also showed that the UM model was successfully established. Compared with the model group, the estrogen concentrations of the serum in the GLC; mifepristone; and high-, medium-, and low- doses of PVE groups were significantly decreased and returned to control levels. Compared with the model group, the progesterone concentrations of the serum were significantly decreased in the high-dose PVE-treated group. These results indicated that the high-dose PVE could decrease the concentrations of estrogen and progesterone.

### Detection of apoptosis in UM

**Fig. 5:**
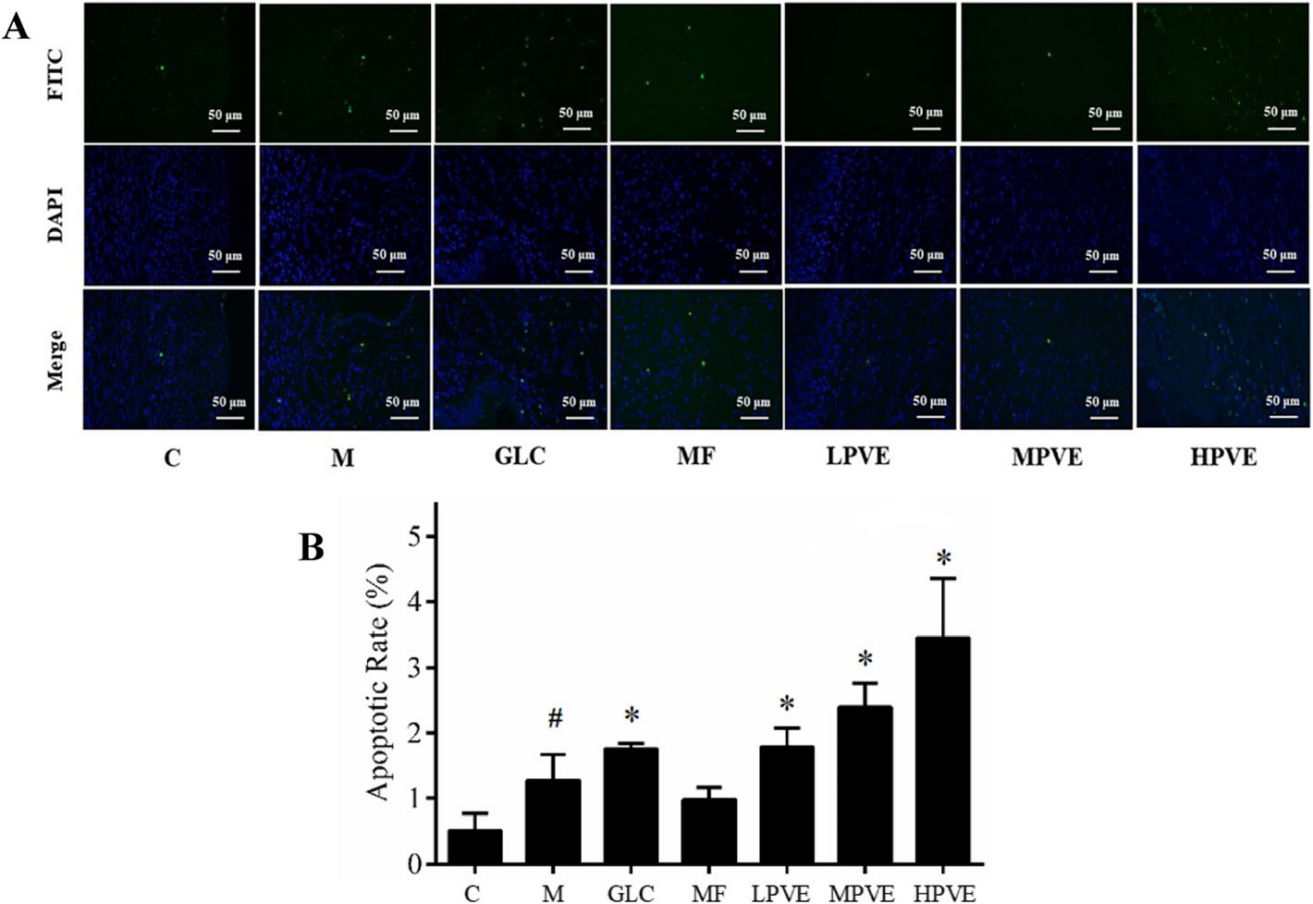
Effects of PVE on apoptosis of the uterus tissue by TUNEL staining. The groups are shown as follows: control (C); model (M); GLC positive control group (GLC, 0.30 g/kg); mifepristone positive control group (MF, 5.4 mg/kg); low-dose PVE (LPVE, 0.11 g/kg); medium-dose PVE (MPVE, 0.22 g/kg); high-dose PVE (HPVE, 0.44 g/kg). **a**: TUNEL staining merged figures, TUNEL (green) staining: the cell apoptosis assay stained in the green channel, DAPI (blue) staining: DAPI was applied as a nuclear counter-stain in the blue channel, × 400 magnification, scale bar:50 μm; **b**: apoptotic rate, compared with the normal group, ^#^*p* < 0.05; compared with the model group, *p < 0.05

The apoptosis in UM was detected by TUNEL staining. As shown in Fig. 5a, greater TUNEL-positive cells in the uterus tissue were found in GLC and PVE treatment groups compared to the model group. As shown in Fig. 5b, the apoptosis index were also significantly increased in GLC and PVE treatment groups compared to the model group. The apoptosis index were increased in high-, and media- doses PVE treatment groups compared to the GLC group. The apoptosis index in PVE treatment groups increased with increasing PVE concentration. These results indicated that PVE could promote the apoptosis of UM cells.

### IHC of UM

**Fig. 6:**
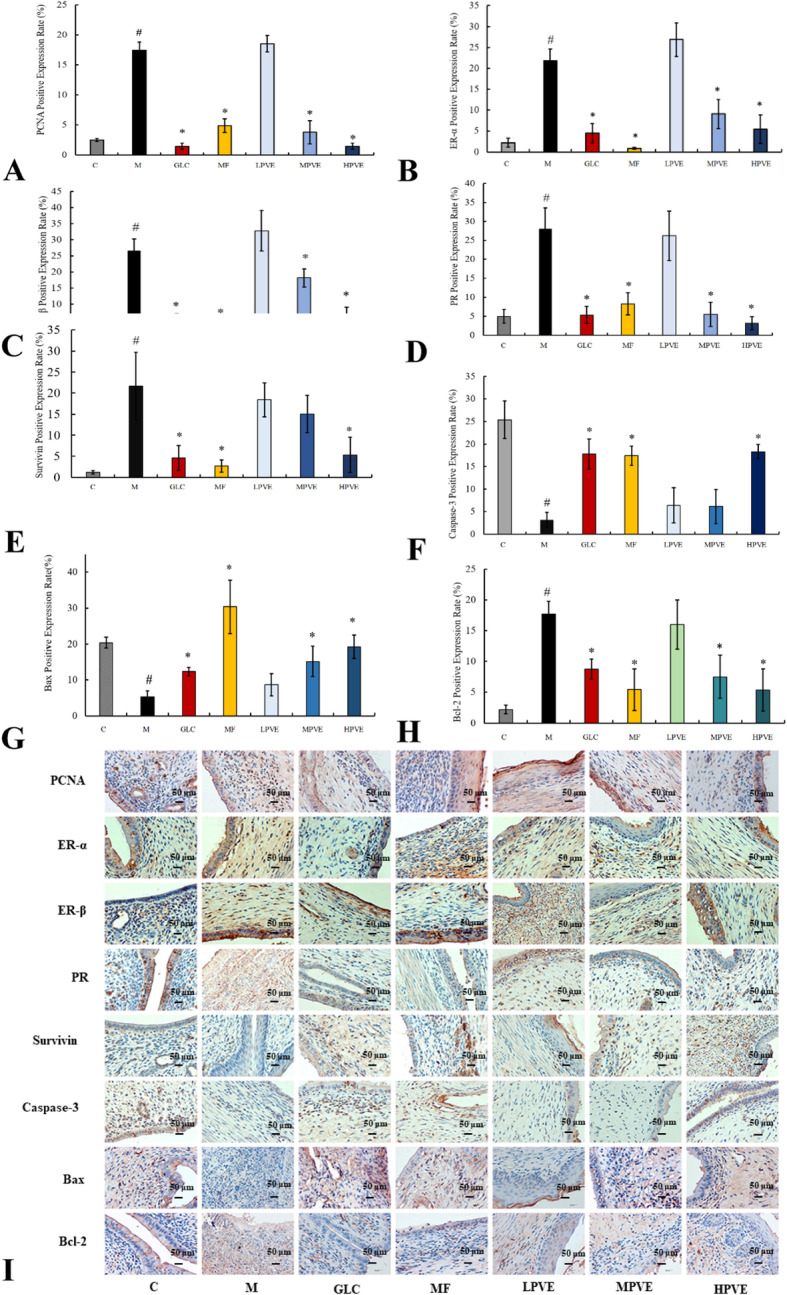
Effects of PVE on positive expression levels of PCNA, ER-α, ER-β, PR, Survivin, Caspase-3, Bax and Bcl-2. The groups are shown as follows: control (C); model (M); GLC positive control group (GLC, 0.30 g/kg); mifepristone positive control group (MF, 5.4 mg/kg); low-dose PVE (LPVE, 0.11 g/kg); medium-dose PVE (MPVE, 0.22 g/kg); high-dose PVE (HPVE, 0.44 g/kg). **a-h**: positive expression rate of PCNA, ER-α, ER-β, PR, Survivin, Caspase-3, Bax, and Bcl-2 in the uterus tissue under PVE treatment by IHC; Compared with the control group, ^#^*P* < 0.05; Compared with the model group, *p < 0.05; **i**: IHC images of PCNA, ER-α, ER-β, PR, Survivin, Caspase-3, Bax, and Bcl-2 in the uterus tissue under PVE treatment by IHC, × 400 magnification, scale bar:50 μm)

Compared with the control group, the model group showed significantly different expression levels of PCNA, PR, ER-α, ER-β, survivin, Bcl-2, caspase-3, and Bax (*p* < 0.05). The expression levels of PCNA, PR, ER-α, ER-β, survivin, and Bcl-2 were downregulated to normal levels after treatment with PVE, while caspase-3 and Bax were upregulated to normal levels after treatment with PVE at the medium and high concentrations. Compared with the model group, the positive control group and the high-dose PVE treatment group showed significant differences in the expression levels of PCNA, PR, ER-α, ER-β, survivin, caspase-3, Bax and Bcl-2 (*p* < 0.05). These results suggest that PVE may induce apoptosis through the downregulation of PCNA, PR, ER-α, ER-β, survivin, and Bcl-2 and the upregulation of caspase-3 and Bax (Fig. 6a-i).

## Discussion

In this study, PVE was extracted by supercritical fluid (CO_2_) extraction with high extraction efficiency and without negative environmental effects. Parameters, such as temperature, pressure and time, that affect the extraction efficiency of PVE, were discussed and optimized by orthogonal testing. Under the optimal conditions, the extraction rate of PVE was 8.1%. Sixteen components, which composed less than 50% of the PVE, were identified by GC-MS. The remaining components of PVE await identification in the future.

Apoptosis is the process of programmed cell death and is generally characterized by distinct changes in morphology and biochemical mechanisms in cells. Apoptosis involves the exposure of phosphatidylserine secondary messengers to the membrane and DNA fragmentation [28]. To confirm the mechanism of PVE-induced UM cell death, flow cytometry analysis was conducted. Early or late apoptosis and DNA damage were detected by Annexin V/PI staining and a PI labeling assay, respectively. The early apoptosis rate of HUMCs treated with PVE (36.59%) was higher than that of the control HUMCs (10.45%). In addition, the late apoptosis rate of HUMCs treated with PVE (20.81%) was higher than that of the control HUMCs (1.71%). PVE induced dose-dependent increases in the total apoptosis rate and decreased the survival rate (Fig. 2).

The cell cycle is closely related to cell proliferation and death, and cell cycle arrest has been used as an indicator of the anticancer activity of a drug. The G1 phase, a stage that occurs before S phase, prepares the cell for division and DNA synthesis [29]. Progression through the cell cycle is controlled by cell cycle checkpoints, i.e., G1, S, G2 and M, and depends on the action of cyclins, cyclin-dependent kinases (CDKs) and cyclin-dependent kinase inhibitors (CDKIs) [30]. Tranilast was found to arrest the proliferation of UM cells at the G0/G1 phase through the suppression of CDK2 activity via an induction of p21^waf1^ and p53 [31]. Growth inhibition and G0/G1 arrest of the cell cycle in NIH3T3 cells was found to be associated with the upregulation of p27 expression and progesterone receptor β [32]. β-Hydroxyisovalerylshikonin-induced apoptosis resulted in an accumulation of the endometriotic stromal cells in the G0/G1 phase of the cell cycle, with a concomitant decrease in the proportion of cells in S phase [33]. The G0/G1 cell population is considered to be an indicator of growth suppression that reflects cell cycle inhibition. In this study, as shown in Table 2, the percentage of cells in G0/G1 stage was higher under PVE treatment than under control treatment. PVE concentration had an effect on G2 phase opposite that observed on G0/G1 phase. These results indicated that PVE promoted the apoptosis of HUMCs, inhibited the proliferation of HUMCs in the G0/G1 stage, and prevented cell entry into the G2 stage for DNA replication, consistent with previous studies. However, further studies was necessary to clarify the the mechanism of PVE-treated UM, which was related to cyclins, cyclin-dependent kinases (CDKs) and cyclin-dependent.

In addition to the increased levels of endogenous and exogenous components in the model group, the incidence of UM was increased in this group. Estrogen and progesterone can promote the proliferation of UM cells. Accordingly, UM has been treated by decreasing the levels of endogenous estrogen and progesterone [34, 35]. In this study, PVE downregulated the levels of estrogen and progesterone in UM, which returned to normal levels (Fig. 4). The apoptosis index was significantly increased in the group treated with the high dose of PVE compared with the model group. These results showed that PVE can induce UM apoptosis. Cyclin PCNA may be related to tumor cell proliferation. Apparent deregulation of PCNA with increased expression in tissues adjacent to tumors has been observed in some breast tumors [36]. The decrease in PCNA expression in PVE-treated UM suggested that the reduction in UM size due to the decreased number of cycling cells was mediated through reductions of ER and PR. The potential mechanism by which PVE inhibits tumor cell proliferation in UM is through decreases in the contents of estrogen and progesterone and downregulation of ER and PR expression through the estrogen signaling pathway.

Bcl-2, which has antiapoptotic properties, is associated with the outer mitochondrial membrane. Bcl-2 stabilizes membrane permeability, thereby preserving mitochondrial integrity, suppressing the release of cytochrome c and inhibiting apoptosis [37]. The Bcl-2 family proteins are the central regulators of mitochondria-mediated apoptosis and are commonly classified into two groups: antiapoptotic members, such as Bcl-2, and proapoptotic members, such as Bax [38]. Our results suggest that PVE may trigger an intrinsic mitochondria-mediated apoptotic pathway by downregulating Bcl-2 expression and upregulating Bax. Survivin is a recently discovered mammalian inhibitor of apoptosis protein and can inhibit Bax-mediated apoptosis. Survivin can inhibit caspase 3 directly and indirectly and thereby prevent apoptosis. Caspase-3 is a primary executioner that catalyzes the cleavage of PARP and actively induces apoptosis [39, 40]. In this study, we found that PVE could downregulate PNCA expression, thereby decreasing the synthesis of caspase-3 and Bax, and inducing apoptosis. The expression levels of PCNA, survivin, caspase-3, Bcl-2, and Bax are related to the mitochondria-mediated apoptotic pathway. The potential apoptotic mechanism of PVE-treated UM is PVE downregulation of PCNA, survivin and Bcl-2 expression and PVE upregulation of caspase-3 and Bax expression through the mitochondria-mediated apoptotic pathway.

## Conclusions

PVE has marked anti-UM activity and is safe and nontoxic. PVE can be used as an ideal candidate drug to treat UM. The potential mechanism, by which PVE inhibits tumor cell proliferation in UM, is through decreases in the contents of estrogen and progesterone and downregulation of ER and PR expression through the estrogen signaling pathway. The potential apoptotic mechanism of PVE-treated UM is PVE downregulation of PCNA, survivin and Bcl-2 expression and PVE upregulation of caspase-3 and Bax expression through the mitochondria-mediated apoptotic pathway.

## Data Availability

All of the data analyzed in this study is included in this article.

## Abbreviations

UM: Uterine Myoma
PVE: Extract of *Prunella vulgaris* L.;
HUSMCs: Human Uterine Smooth Muscle Cells
HUMCs: Human Uterine Myoma Cells
MTT: 3-(4,5-Dimethylthiazol-2-yl)-2,5-Diphenyltetrazolium Bromide
DMEM: Dulbecco’s Modified Eagle Medium
PBS: Phosphate-Buffered Saline
GLC: Gong Liu-Qing Capsule
ELISA: Enzyme-Linked Immunosorbent Assay
HE: Hematoxylin and Eosin Staining
ER-α: Estrogen Receptor Alpha
ER-β: Estrogen Receptor Beta
PR: Progesterone Receptor
IHC: Immunohistochemistry.
